# The insertion of the inverted repeat of an insertion sequence (IS) element from *Lacticaseibacillus rhamnosus* changes the host range and stability of pGK12, a shuttle vector for lactic acid bacteria

**DOI:** 10.1128/aem.01908-24

**Published:** 2025-03-14

**Authors:** Zifan Xie, Yong-Su Jin, Todd R. Klaenhammer, Michael J. Miller

**Affiliations:** 1 Food Science and Human Nutrition, University of Illinois Urbana-Champaign 14589 , Urbana, Illinois, USA; 2 Carl R. Woese Institute for Genomic Biology, University of Illinois Urbana-Champaign 14589 , Urbana, Illinois, USA; 3 Department of Food, Bioprocessing and Nutrition Sciences, North Carolina State University 6798 , Raleigh, North Carolina, USA; Anses, Maisons-Alfort Laboratory for Food Safety, Maisons-Alfort, France

**Keywords:** *Lactobacillus*, IS element, plasmids, plasmid replication, heterologous gene expression

## Abstract

Insertion sequences (ISs) are key components of most bacterial genomes and play a crucial role in bacterial mutagenesis. In this study, we observed the insertion of an IS element, ISLrh, from the *Lacticaseibacillus rhamnosus* M1 genome into plasmid pGK12, resulting in the generation of a new plasmid, pTRK829. This insertion enabled pTRK829 to replicate in hosts previously incompatible with pGK12, including *L. rhamnosus* M1, *L. rhamnosus* GG (LGG), *Lacticaseibacillus casei* ATCC 393, and *Lacticaseibacillus paracasei* ATCC 25598. However, the ISLrh-inserted plasmid, pTRK829, was unstable and underwent a spontaneous deletion, resulting in a smaller and more stable variant, pTRK830, which retained ISLrh. Characterization of pTRK829 and pTRK830 across several host strains showed that ISLrh insertion led to a dramatic alteration in host range and impacted plasmid stability and copy number. Sequence and functional analysis of pTRK830 revealed that the terminal inverted repeats (IRs) of the inserted ISLrh and its insertion location were essential for plasmid replication in LGG. Finally, pTRK830 was successfully used as an expression vector for heterologous β-glucuronidase expression in LGG, *L. casei* ATCC 393, and *L. paracasei* ATCC 25598. In conclusion, this study demonstrated that the insertion of the IRs from ISLrh at a specific location can directly change the host range and stability of pGK12. Furthermore, we also demonstrated the potential of pTRK830 as a new cloning and expression vector for genetically intractable lactobacilli.

**IMPORTANCE:**

This study highlights the significant impact of insertion sequence (IS) elements on plasmid replication in lactobacilli. The spontaneous integration of an IS element from the *Laticaseibacillus rhamnosus* genome into plasmid pGK12 not only expands its host range in previously incompatible strains but also changes plasmid stability and copy number. This expansion of the plasmid's host range is crucial for developing versatile genetic tools across diverse lactobacilli species. Additionally, the stable plasmid variant of pGK12 with the IS element insertion offers a valuable tool for cloning and gene expression in lactobacilli. These findings enhance our understanding of plasmid-IS element interactions and may provide insight into a new method to expand the host range of existing plasmids.

## INTRODUCTION

Lactobacilli play a crucial role in numerous beneficial applications, including probiotics, industrial starter cultures, and production microorganisms for various value-added compounds and food ingredients. Due to their health benefits and generally recognized as safe (GRAS) status, numerous lactobacilli are considered ideal vehicles for genetic engineering in industrial and therapeutic applications (1, 2). Successful genetic engineering in lactobacilli requires efficient cloning vectors to introduce, modify, or remove target genes. Broad-host-range plasmids are of considerable interest because they can stably replicate in bacteria from different phylogenetic subgroups (3).

**Fig 1:**
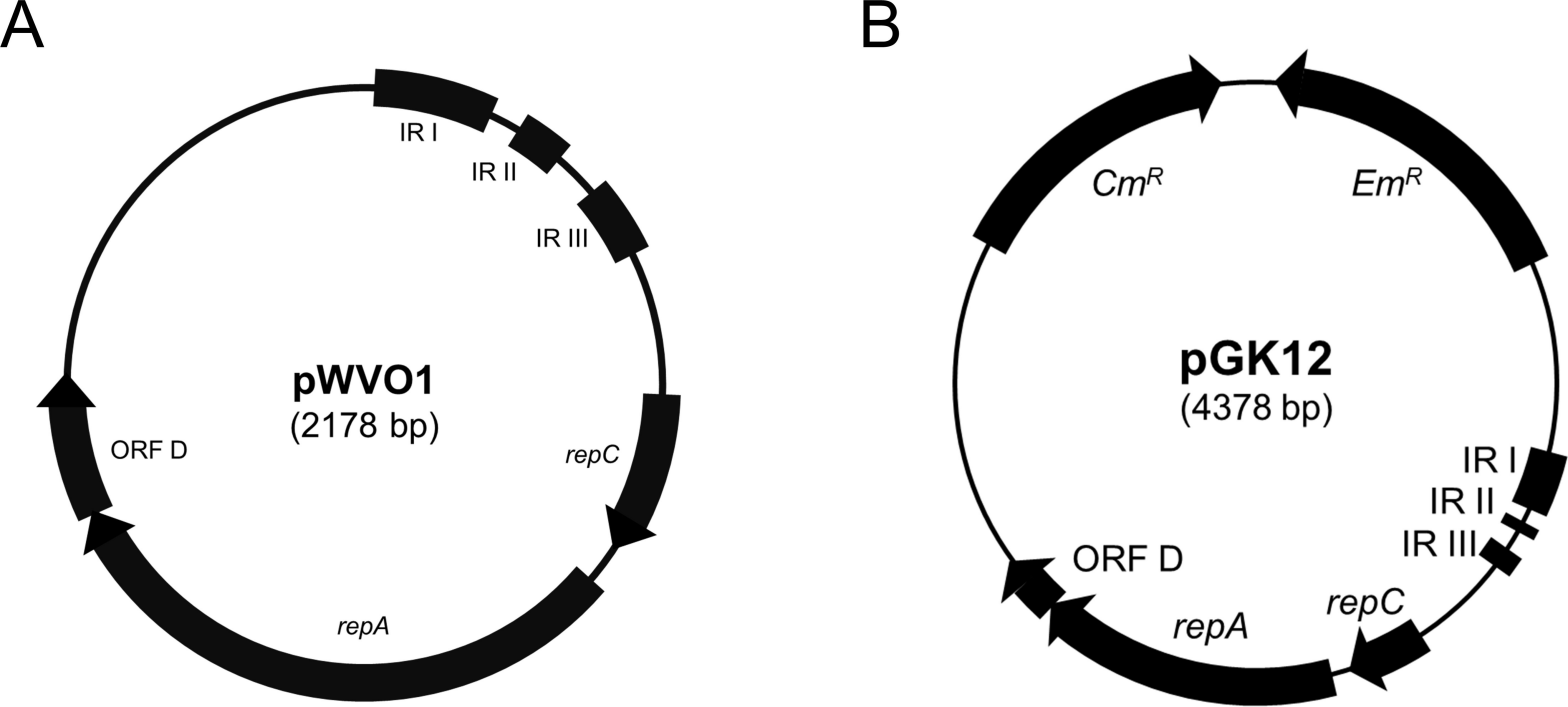
Genetic maps of (**A**) pWVO1 and (**B**) pGK12.

There are three general replication mechanisms for circular plasmids, namely, theta-type, rolling-circle, and strand displacement-type replication (4, 5). Most plasmids isolated from Gram-positive bacteria replicate via rolling circle replication (RCR). These plasmids contain a single-stranded origin (*sso*), a double-stranded origin (*dso*), and genes for their own replication proteins (Rep). One example of an RCR plasmid is pWVO1 (Fig. 1A), originally isolated from *Lactococcus lactis* subsp. *cremoris* (6, 7). pWVO1 has a *dso* in the inverted repeat (IR) region III, an *sso* in the IR regions I and II, a *repA* gene encoding the replication protein, and a *repC* gene coding for a 6 kDa protein that negatively regulates *repA* expression (7, 8). Additionally, there is another open reading frame (ORF) called ORFD, which is putatively involved in copy number regulation but is not essential for replication (7). The RCR origin of pWVO1 has been used to create many cloning vectors (9–11). Among them, pGK12 (Fig. 1B), constructed by inserting the chloramphenicol resistance gene (*Cm^R^*) and erythromycin resistance gene (*Em^R^*) into pWVO1 (9), is one of the most widely used plasmids in lactobacilli genetic engineering. Like pWVO1, pGK12 also replicates in a wide variety of Gram-positive bacteria (such as lactobacilli, lactococci, streptococci, clostridia, and staphylococci) as well as Gram-negative bacteria, including *Escherichia coli*. However, pGK12 is known to be unstable and does not replicate to a high copy number in these species (9, 11).

*Lacticaseibacillus rhamnosus* is a diverse species, with strains isolated from various ecological niches (12, 13). Genetic rearrangements caused by insertion sequence (IS) elements have greatly contributed to *L. rhamnosus* niche adaptation (14, 15). IS elements are the simplest mobile genetic elements (MGEs) and are widely distributed across bacterial genomes (16, 17). These transposable elements consist of a transposase gene, which catalyzes their transposition, and are flanked by IRs (18). IS elements can exist in multiple copies within a particular genome and have the ability to move within or between genomes as part of other MGEs such as phages and plasmids (19). IS elements significantly contribute to the occurrence of mutations, gene inactivation, gene overexpression, and chromosomal rearrangements (16, 17, 19). Moreover, IS elements are valuable in biotechnology applications (20) and bacterial strain typing (21).

Despite its broad host range, no studies have demonstrated pGK12 replication in *L. rhamnosus*. In this study, we observed that the unique insertion of an IS element from the *L. rhamnosus* M1 genome into pGK12 enabled the resulting plasmids to stably replicate in *L. rhamnosus* and closely related species, which were previously untransformable with pGK12. Furthermore, IS element insertion also altered the stability and copy number of plasmids in several lactobacilli strains. Further analysis revealed that these changes were closely related to the IRs of the inserted IS element and its specific insertion location. Additionally, we demonstrated the potential of the IS element-inserted plasmid, pTRK830, as an effective cloning and expression vector for various biotechnological applications in genetically intractable lactobacilli.

## RESULTS

### Insertion of an IS element into pGK12 resulting in replicable plasmids in *L. rhamnosus*

**Fig 2:**
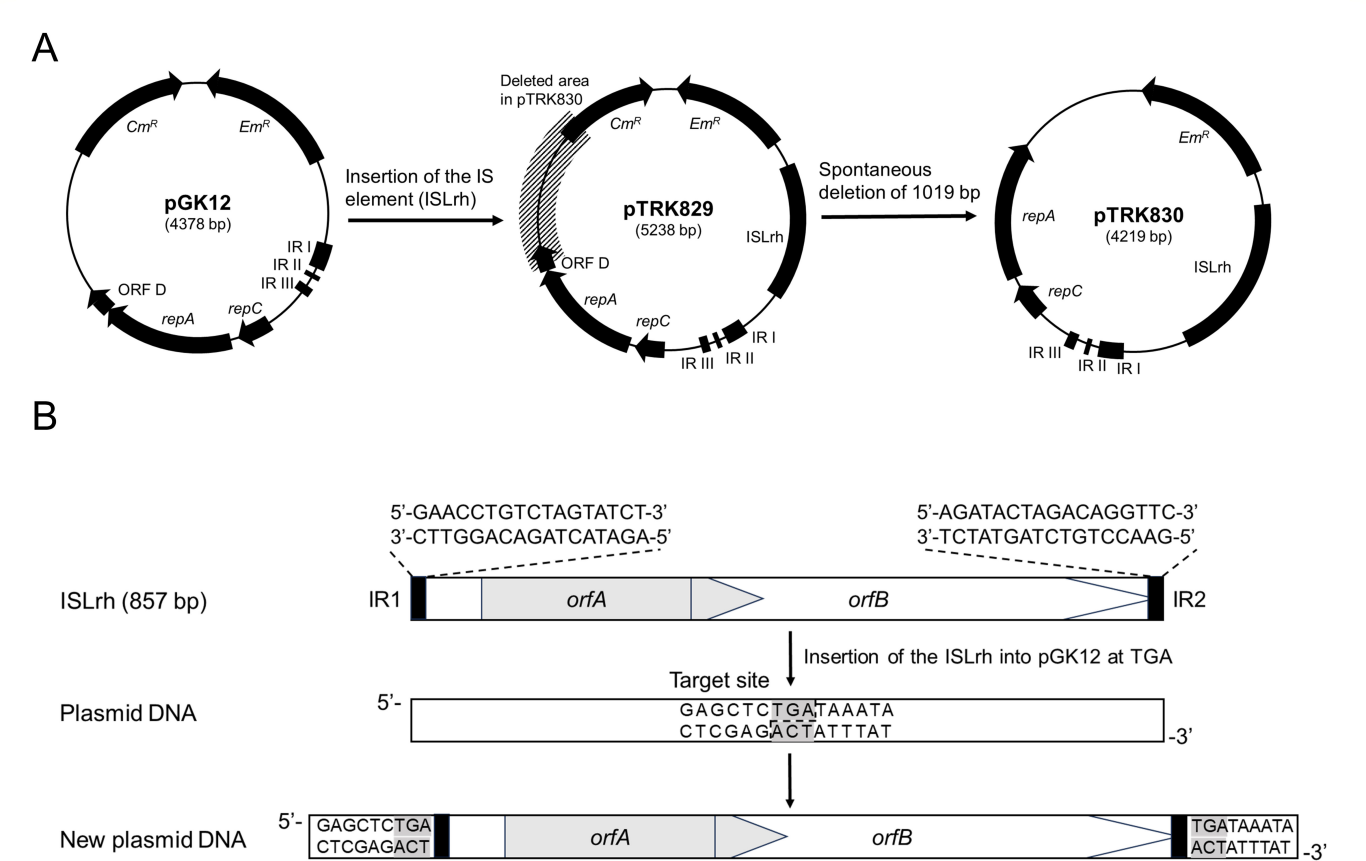
(**A**) Schematic diagram of the discovery process of plasmids pTRK829 and pTRK830. An IS element (ISLrh) from *L. rhamnosus* M1 was spontaneously inserted into pGK12, generating a new plasmid, designated pTRK829. Examination of pTRK829 *L. rhamnosus* M1 transformants identified a derivative, designated pTRK830. pTRK830 revealed that it retained ISLrh but had a 1,019 bp deletion (the shadow area) in the plasmid resulting in the loss of a portion of the *Cm^R^* gene and ORFD. (**B**) The insertion of ISLrh into pGK12. ISLrh is flanked by two 17 bp terminal IRs (black boxes). ISLrh contains two partially overlapping ORFs, *orfA* (gray box) and *orfB* (white box). The insertion of ISLrh created 3 bp target repeats at the insertion site 5′-TGA-3′ (highlighted in gray). The diagram is not to scale.

After numerous unsuccessful attempts to transform *L. rhamnosus* M1 with pGK12, we successfully recovered a single transformant harboring a plasmid larger than pGK12. This plasmid, characterized as pGK12 with an 860 bp insert, was designated pTRK829 (Fig. 2A). The transformation efficiency (transformants per microgram plasmid DNA) of pTRK829 into *L. rhamnosus* M1 was 10^3^–10^4^ CFU/µg (Table 1), whereas repeated experiments with native pGK12 failed to yield additional transformants.

**TABLE 1:** Host range and transformation efficiency (CFU/μg DNA) of pGK12 and its variants

		pGK12	pTRK829	pTRK830
*L. rhamnosus* M1		0	1.1 × 10^4^	8.0 × 10^3^
*L. rhamnosus* GG, ATCC 53103		0	2.1 × 10^4^	2.9 × 10^4^
*L. casei* ATCC 393		0	2.3 × 10^2^	3.4 × 10^2^
*L. paracasei* ATCC 25598		0	1.2 × 10^4^	1.8 × 10^4^
*L. paracasei* ATCC 334		8.0 × 10^1^	2.4 × 10^3^	1.1 × 10^4^
*L. gasseri* ATCC 33323		5.0 × 10^4^	4.5 × 10^4^	5.9 × 10^4^
*L. johnsonii* ATCC 11506		2.5 × 10^4^	6.2 × 10^3^	3.1 × 10^4^
*L. paraplantarum* ATCC 700211		7.5 × 10^4^	3.9 × 10^3^	6.3 × 10^3^
*L. plantarum* ATCC 14917		2.5 × 10^4^	0	0
*L. fermentum* ATCC 9338		1.4 × 10^4^	0	0
*L. acidophilus* NCFM		3.4 × 10^3^	0	0

Sequence analysis of the insert in plasmid pTRK829 identified an IS element, designated ISLrh, which was integrated 282 bp upstream of the *sso* (Fig. 2A). ISLrh is 857 bp in length and belongs to the IS1031 subgroup in the IS5 family. It contains two 17 bp terminal IRs with the sequence 5′-GAACCTGTCTAGTATCT-3′ (Fig. 2B). Like some other members of the IS1031 subgroup (16), ISLrh contains two partially overlapping ORFs, *orfA* and *orfB*. A −1 translational frameshift results in a full-length transposase (GenBank accession number: QFG48548.1). Members of the IS1031 group exhibit a preference for insertion sites with the sequence 5′-TNA-3′ and generate an exclusive 3 bp direct repeat, which matches the insertion site in pGK12 (5′-TGA-3′; Fig. 2B) (16).

Further examination of pTRK829 *L. rhamnosus* M1 transformants revealed a new variant, designated pTRK830. While pTRK830 retained the 860 bp ISLrh insertion, it had a 1,019 bp deletion elsewhere in the plasmid, resulting in the loss of a part of the *Cm^R^* gene and ORFD (Fig. 2A). pTRK830 exhibited the same transformation efficiencies as pTRK829 in *L. rhamnosus* M1 (Table 1).

### Changes in plasmid host range due to ISLrh insertion

To determine whether the insertion of ISLrh changed the host range of plasmids, we tested the replication capability of pGK12 and its variants, pTRK829 and pTRK830, in 10 widely used lactobacilli strains (Table 1).

In addition to *L. rhamnosus* M1, ISLrh-inserted plasmids, pTRK829 and pTRK830, were successfully introduced and could replicate in three other strains, *L. rhamnosus* GG (LGG), *Lacticaseibacillus casei* ATCC 393, and *Lacticaseibacillus paracasei* ATCC 25598, which could not be transformed with pGK12. On the other hand, *L. paracasei* ATCC 334, *Lactobacillus gasseri* ATCC 33323, *Lactobacillus johnsonii* ATCC 11506, and *Lactiplantibacillus paraplantarum* ATCC 700211 were successfully transformed with both pGK12 and its variants. Notably, in *L. paracasei* ATCC 334, the insertion of ISLrh resulted in a noticeable increase in transformation efficiency, rising from approximately 10^1^ CFU/µg with pGK12 to 10^3^–10^4^ CFU/µg with either pTRK829 or pTRK830 (Table 1). Furthermore, the transformation efficiency of pGK12 and its variants in *L. gasseri* ATCC 33323, *L. johnsonii* ATCC 11506, and *L. paraplantarum* ATCC 700211 was similar, ranging from 10³−10⁴ CFU/µg (Table 1). However, pTRK829 and pTRK830 transformants were not obtained with *Lactiplantibacillus plantarum* ATCC 14917, *Limosilactobacillus fermentum* ATCC 9338, and *Lactobacillus acidophilus* NCFM, despite these strains being successfully transformed with pGK12 (Table 1).

Overall, these results suggest that the insertion of ISLrh into pGK12 changes the plasmid’s host range, enabling it to transform additional strains while losing the ability to transform others.

### Segregational stability of pGK12 and its variants

**Fig 3:**
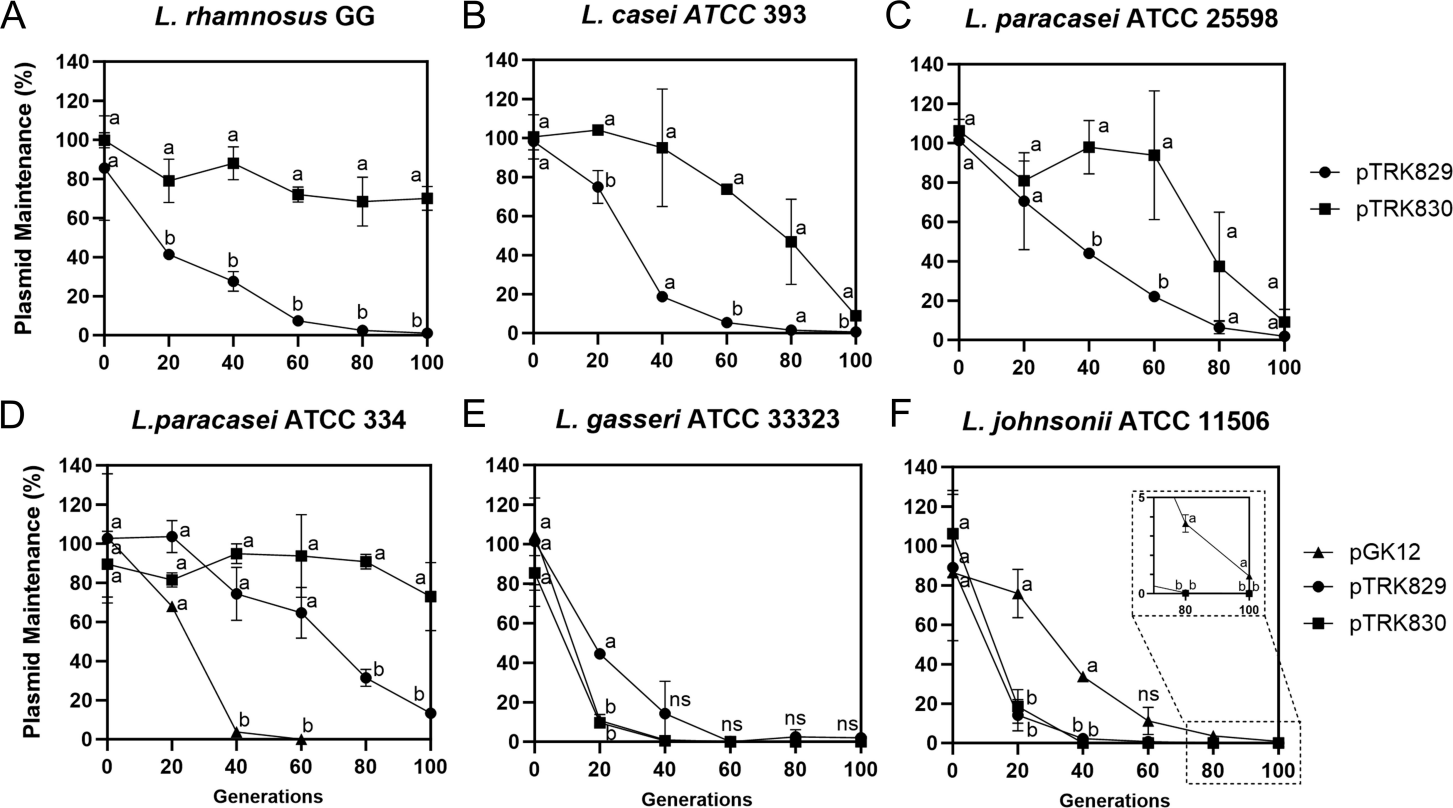
Plasmid stability of pGK12 and ISLrh-inserted plasmids in six host strains. The plasmid stability experiments were conducted in the following strains: (**A**) LGG, (**B**) *L. casei* ATCC 393, (**C**) *L. paracasei* ATCC 25598 with pTRK829 and pTRK830, (**D**) *L. paracasei* ATCC 334, (**E**) *L. gasseri* ATCC 33323, and (**F**) *L. johnsonii* ATCC 11506 with pGK12, pTRK829, and pTRK830. Plasmid stability was assessed by plating on de-Man-Rogosa-Sharpe (MRS) agar with and without erythromycin to determine viable cell counts after repeated cultivation in MRS broth. Plasmid maintenance (%) was calculated as the ratio of CFUs on selective vs non-selective MRS plates. Data are presented as means ± SD from at least two biological replicates. Distinct letters are employed to denote significant differences (*P <* 0.05) at the same generation. “ns” indicates no significant difference between groups.

The segregational stability of pTRK829 and pTRK830 was determined in select hosts, LGG, *L. casei* ATCC 393, and *L. paracasei* ATCC 25598, which could be transformed with these variants but not with pGK12. Meanwhile, the plasmid stability of pGK12 and its variants was tested in *L. paracasei* ATCC 334, *L. gasseri* ATCC 33323, and *L. johnsonii* ATCC 11506, which were able to be transformed with both pGK12 and its variants. The results are summarized in Fig. 3.

Across the host strains LGG, *L. casei* ATCC 393, and *L. paracasei* ATCC 25598, plasmid pTRK829 was poorly maintained under non-selective conditions (Fig. 3A through C). In contrast, the spontaneous mutant vector pTRK830, featuring a 1,019 bp deletion, was more stable than pTRK829 in these host strains (Fig. 3A through C). These results suggest a potential correlation between plasmid instability and the size of the RCR plasmids.

Notably, the impact of ISLrh insertion on plasmid segregational stability varies across the host strains *L. paracasei* ATCC 334, *L. gasseri* ATCC 33323, and *L. johnsonii* ATCC 11506, which can be transformed with both pGK12 and its variants. In *L. paracasei* ATCC 334, the parental plasmid pGK12 was extremely unstable, while the insertion of ISLrh improved its segregational stability (Fig. 3D). Conversely, in *L. gasseri* ATCC 33323, ISLrh insertion had no effect on plasmid stability, with all three plasmids being extremely unstable (Fig. 3E). Interestingly, in *L. johnsonii* ATCC 11506, the parental plasmid pGK12 was slightly more stable than its ISLrh-inserted variants (Fig. 3F).

### Growth rate of host strains bearing pGK12 and its variants

During the stability experiment, we observed potential growth differences, suggesting that the vectors may impose a significant metabolic burden on the host strains. To evaluate this, we determined the maximum growth rate of pGK12, pTRK829, and pTRK830 transformants of *L. paracasei* ATCC 334, *L. gasseri* ATCC 33323, and *L. johnsonii* ATCC 11506 (Table 2).

**TABLE 2:** Maximum growth rate (h^−1^)^*a*^

	No plasmid	pGK12	pTRK829	pTRK830
*L. paracasei* ATCC 334	0.1067 ± 0.0035^a^	0.0690 ± 0.0013^c^	0.0854 ± 0.0015^b^	0.0732 ± 0.0022^c^
*L. gasseri* ATCC 33323	0.0421 ± 0.0032^a^	0.0433 ± 0.0016^a^	0.0616 ± 0.0145^a^	0.0495 ± 0.0092^a^
*L. johnsonii* ATCC 11506	0.0957 ± 0.0016^a^	0.0901 ± 0.0025^ab^	0.0789 ± 0.0093^bc^	0.0718 ± 0.0013^c^

^*a*^ Data are presented as means ± SDs from three biological replicates. Distinct superscript letters (a, b, c) are employed to denote significant differences (*P <* 0.05) in the same row.

**Fig 4:**
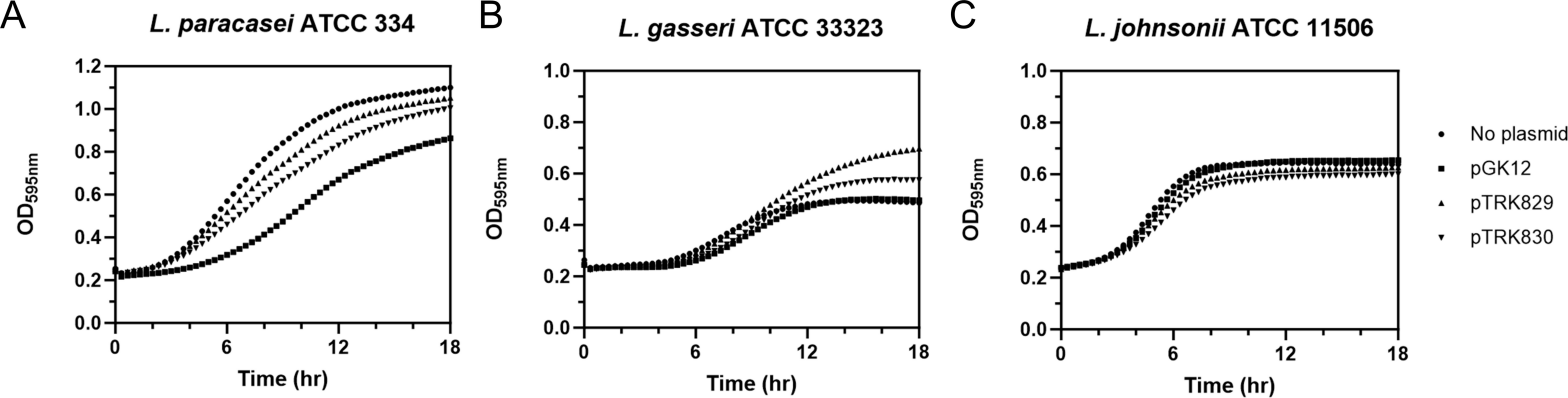
Growth curves of (**A**) *L. paracasei* ATCC 334, (**B**) *L. gasseri* ATCC 33323, and (**C**) *L. johnsonii* ATCC 11506 without plasmid or with plasmid pGK12, pTRK829, or pTRK830 under non-selective conditions. Data represent averages from three biological replicates. Error bars are not shown for clarity.

Under non-selective conditions, *L. paracasei* ATCC 334 with no plasmid had the fastest growth rate (µ_max, no plasmid_ = 0.1067 ± 0.0035 h^−1^). The strain with pGK12 (µ_max, pGK12_ = 0.0690 ± 0.0013 h^−1^) grew significantly slower than that with pTRK829 (µ_max, pTRK829_ = 0.0854 ± 0.0015 h^−1^, *P <* 0.05). Further inspection of the growth curves (Fig. 4A) determined that *L. paracasei* ATCC 334 with pGK12 exhibited a longer lag time and a lower final OD_595nm_ compared to the strain with pTRK830, despite having similar maximum growth rates (Table 2). These results indicate that, under nonselective conditions, pGK12 imposes a higher plasmid burden on *L. paracasei* ATCC 334 compared to the pTRK829 and pTRK830 plasmids, suggesting that the insertion of ISLrh may reduce the plasmid metabolic burden in this host.

Beyond reducing the plasmid burden in *L. paracasei* ATCC 334, the effects of ISLrh insertion on the growth rate of pGK12 differ among other host strains, such as *L. gasseri* ATCC 33323 and *L. johnsonii* ATCC 11506. In *L. gasseri* ATCC 33323, there were no significant growth rate differences between plasmid-bearing and non-plasmid-bearing strains under non-selective conditions (Table 2; Fig. 4B). For *L. johnsonii* ATCC 11506, the strain without plasmids exhibited the highest maximum growth rate (µ_max, no plasmid_ = 0.0957 ± 0.0016 h^−1^). No significant reduction was observed for pGK12 (µ_max, pGK12_ = 0.0901 ± 0.0025 h^−1^, *P =* 0.54), but significant reductions were observed for the ISLrh-inserted variants pTRK829 (µ_max, pTRK829_ = 0.0789 ± 0.0093 h^−1^, *P <* 0.05) and pTRK830 (µ_max, pTRK830_ = 0.0718 ± 0.0013 h^−1^, *P <* 0.05) when compared to the no plasmid control. This suggests that the insertion of ISLrh into pGK12 potentially imposes a growth burden in *L. johnsonii* ATCC 11506 (Table 2; Fig. 4C).

### Plasmid copy number of pGK12 and its variants

We then assessed the plasmid copy numbers (PCNs) in these six strains, LGG, *L. casei* ATCC 393, *L. paracasei* ATCC 25598, *L. paracasei* ATCC 334, *L. gasseri* ATCC 33323, and *L. johnsonii* ATCC 11506. PCNs were determined by comparing the genomic *groEL* gene with the plasmid-borne *Em^R^* gene using real-time quantitative PCR (qPCR) under selective conditions (22). The results are summarized in Table 3.

**TABLE 3:** Plasmid copy number (PCN) in lactobacilli (copies per chromosome)^*a*^

	pGK12	pTRK829	pTRK830
LGG	N/A	2.59 ± 1.82^a^	3.42 ± 0.98^a^
*L. casei* ATCC 393	N/A	25.19 ± 5.57^b^	40.19 ± 3.25^a^
*L. paracasei* ATCC 25598	N/A	9.85 ± 3.20^a^	13.40 ± 5.24^a^
*L. paracasei* ATCC 334	<1.00^b^	4.20 ± 2.24^a^	8.62 ± 3.33^a^
*L. gasseri* ATCC 33323	48.99 ± 4.72^a^	36.90 ± 20.31^a^	57.72 ± 14.97^a^
*L. johnsonii ATCC* 11506	4.88 ± 1.64^c^	50.85 ± 10.02^a^	27.77 ± 7.32^b^

^*a*^ Data are presented as means ± SD from three biological replicates. Distinct superscript letters (a, b, c) are employed to denote significant differences (*P <* 0.05) in the same row. N/A indicates data not applicable, because plasmid pGK12 could not be transformed into the strain.

The data reveal large variations in PCNs among the tested lactobacilli species and plasmids. Specifically, both pTRK829 and pTRK830 are characterized as low-copy number plasmids (five or fewer copies per cell) in LGG, high-copy number plasmids (20 or more copies per cell) in *L. casei* ATCC 393, and medium-copy number plasmids (~10 copies per cell) in *L. paracasei* ATCC 25598. In *L. paracasei* ATCC 334, consistent with the transformation and stability results, the insertion of ISLrh also led to an increase in PCNs, with pTRK829 and pTRK830 showing significantly higher PCNs than pGK12. Interestingly, across all four strains mentioned above, LGG, *L. casei* ATCC 393, *L. paracasei* ATCC 25598, and *L. paracasei* ATCC 334, the more stable plasmid pTRK830 also exhibited a higher copy number than pTRK829. Furthermore, in *L. gasseri* ATCC 33323, although all three plasmids were unstable, they were characterized as high-copy number plasmids in this host strain. Lastly, in *L. johnsonii* ATCC 11506, the ISLrh-inserted plasmids (pTRK829 and pTRK830) had significantly higher copy numbers compared to the parental plasmid pGK12.

### Essential factors of ISLrh for enabling plasmid replication in *L. rhamnosus*

To study how the insertion of ISLrh enabled plasmid pGK12 to gain replication ability in *L. rhamnosus*, we applied frameshift mutations and a deletion strategy to ISLrh region in pTRK830 and performed reverse engineering with pGK12. These approaches allowed us to identify the essential part of ISLrh required for plasmid replication in *L. rhamnosus*. We selected pTRK830 as the study objective due to its greater stability compared to pTRK829. LGG was chosen as the host strain because it is one of the most characterized and studied probiotic strains with a fully sequenced genome (23, 24).

**Fig 5:**
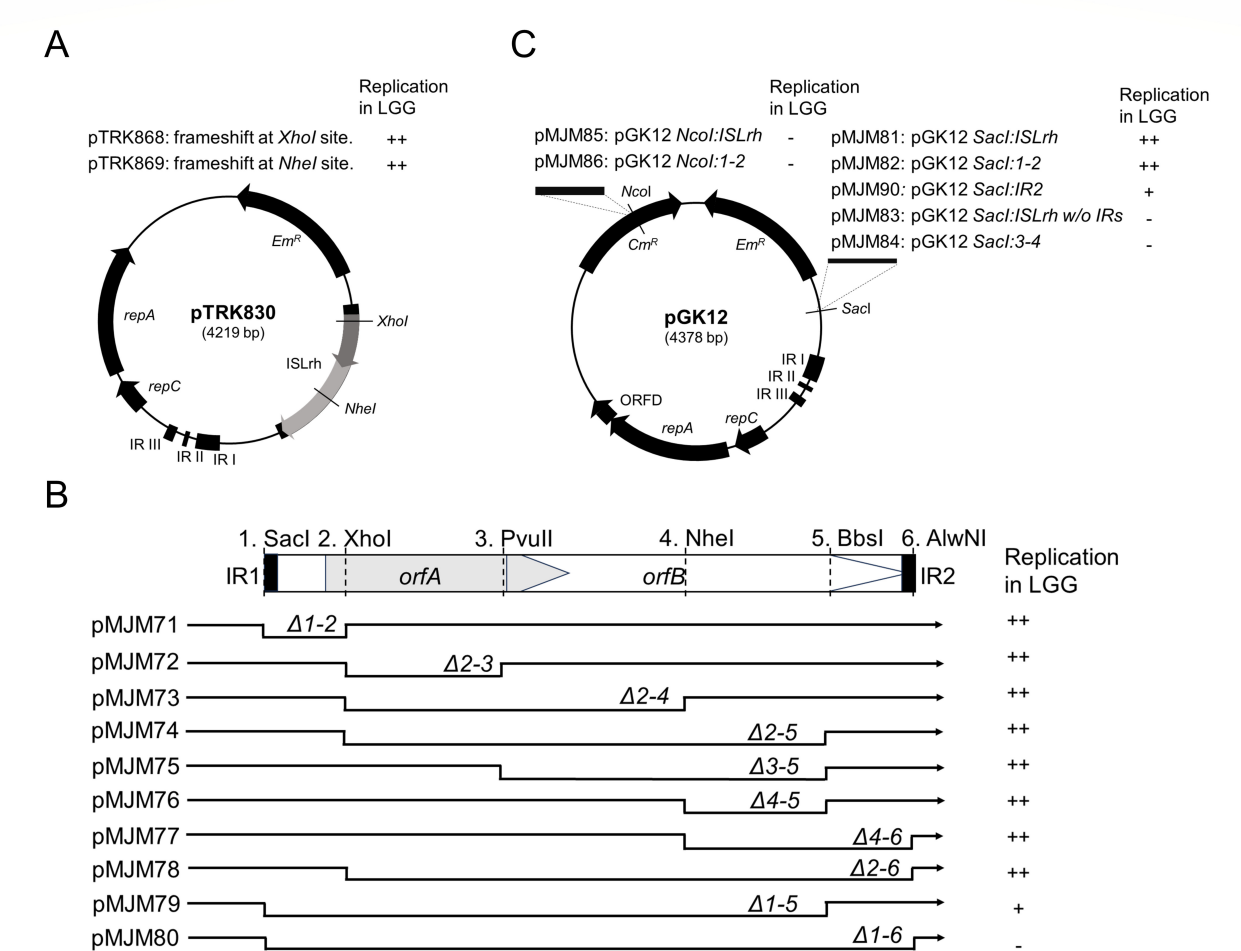
Effects of ISLrh on plasmid replication. (**A**) Frameshift mutations in *orfA* or *orfB*. (**B**) Location of deletions in ISLrh in pTRK830 and their effects on plasmid replication in LGG. The relative location and the direction of transcription of each ORF in pTRK830 are indicated by horizontal arrows. The IRs are indicated by black bars. The names of deletion plasmids are indicated. (**C**) Sequences of the inserts and their insertion locations in pGK12 and their effects on plasmid replication in LGG. Thick black lines represent the fragments cloned into pGK12. The names of recombinant plasmids containing these inserts are indicated. “−” indicates no transformant; “+” indicates transformation efficiency is ~10^1^ CFU/µg DNA; “++” indicates transformation efficiency is 10^3^–10^5^ CFU/µg DNA.

The first hypothesis we tested was that the transposase activity of ISLrh was necessary for the replication of pTRK830 in LGG. To investigate this, we created frameshift mutations in either *orfA* or *orfB*, generating pTRK868 and pTRK869, respectively (Fig. 5A). The results showed that these mutations did not alter transformation efficiency, suggesting that a functioning transposase is not necessary for the replication of pTRK830 in LGG.

**Fig 6:**
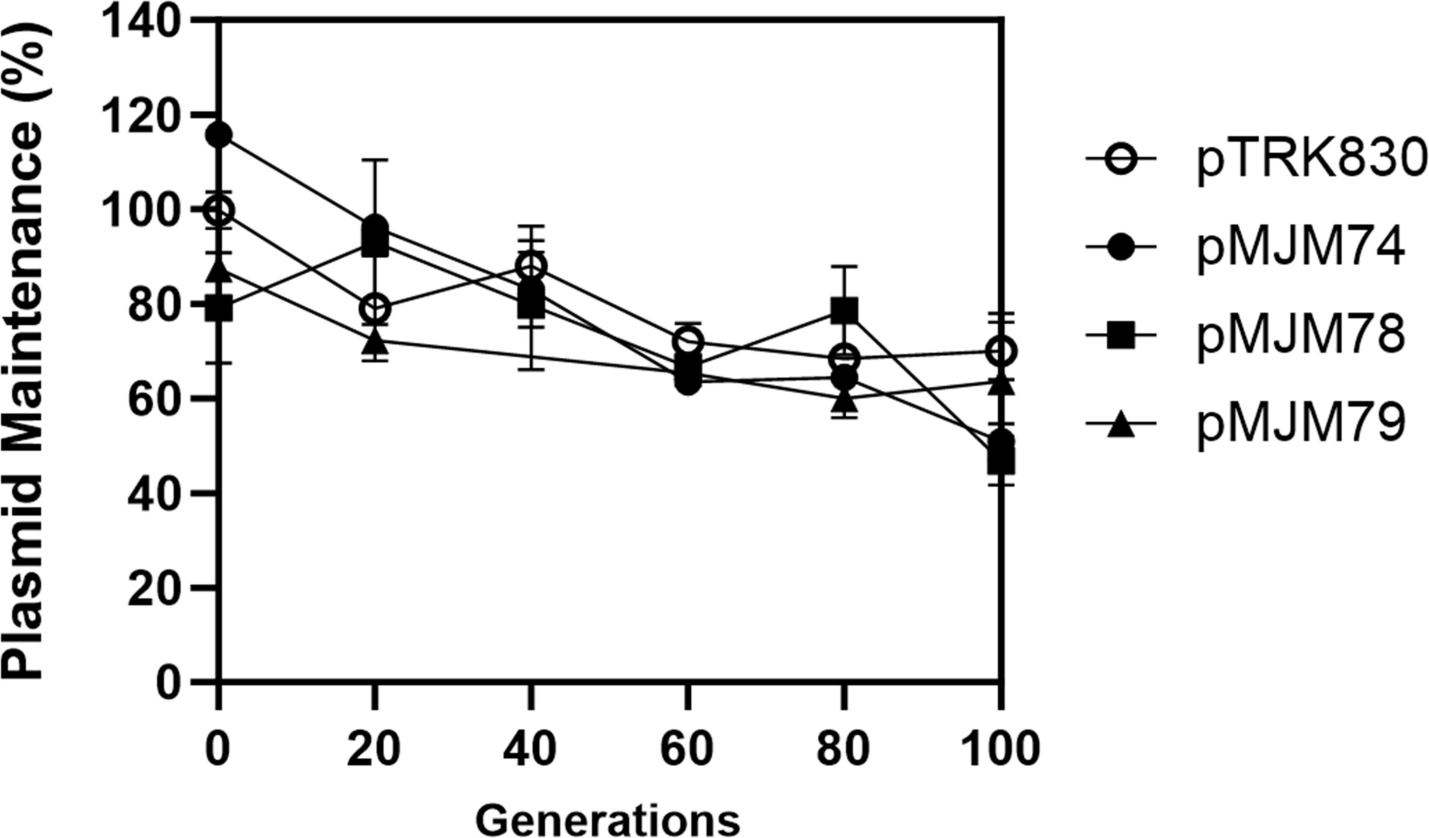
Stability of pTRK830 and its deletion derivative plasmids in LGG. Data are presented as means ± SD from at least two biological replicates.

Then, we hypothesized that there was an essential region in ISLrh that activated the replication ability of pTRK830 in LGG. To localize the essential region, we initially applied a deletion strategy to pTRK830 (Fig. 5B). By deleting different parts of ISLrh in pTRK830, we generated plasmids pMJM71-80 (Table 4). The deletion plasmids were confirmed by sequencing. Transformation of these deletion plasmids into LGG yielded high transformation efficiency (10^4^–10^5^ CFU/µg) for plasmids pMJM71-78. However, the transformation efficiency for pMJM79 was extremely low (10^1^–10^2^ CFU/µg), and no transformants were observed with pMJM80, in which the entire ISLrh was deleted, leaving only a 2 bp sequence preceding the 3 bp insertion site (5′-GCTGA-3′). Furthermore, we studied the segregational stability of pTRK830 and its deletion derivatives (Fig. 6). Results showed that the ISLrh partially deleted plasmids had similar stability as pTRK830, indicating that the integrity of ISLrh does not affect plasmid stability. Overall, these results suggest that the IRs of ISLrh are the essential region for plasmid replication and stability in LGG.

**TABLE 4:** Plasmids used in this study

Plasmid	Description	Source or reference
pGK12	ori (pWVO1), Cm^R^, Em^R^, and Gram-positive shuttle vector	(9)
pMJM28	Em^R^, derivative of pGK12, *gusA3* expressing vector	Our lab
pTRK829	Cm^R^ and Em^R^, derivative of pGK12, contains ISLrh between *Em^R^* and *sso*	This study
pTRK830	Em^R^, derivative of pTRK829, contains ISLrh between *Em^R^* and *sso*	This study
pTRK868	Em^R^, derivative of pTRK830, frameshift in *orf*A	This study
pTRK869	Em^R^, derivative of pTRK830, frameshift in *orf*B	This study
pMJM71	Em^R^, made from pTRK830 by removing the 1–2 (SacI-XhoI) fragment from ISLrh	This study
pMJM72	Em^R^, made from pTRK830 by removing the 2–3 (XhoI-PvuII) fragment from ISLrh	This study
pMJM73	Em^R^, made from pTRK830 by removing the 2–4 (XhoI-NheI) fragment from ISLrh	This study
pMJM74	Em^R^, made from pTRK830 by removing the 2–5 (XhoI-BbsI) fragment from ISLrh	This study
pMJM75	Em^R^, made from pTRK830 by removing the 3–5 (PvuII-BbsI) fragment from ISLrh	This study
pMJM76	Em^R^, made from pTRK830 by removing the 4–5 (NheI-BbsI) fragment from ISLrh	This study
pMJM77	Em^R^, made from pTRK830 by removing the 4–6 (NheI-AlwNI) fragment from ISLrh	This study
pMJM78	Em^R^, made from pTRK830 by removing the 2–6 (XhoI-AlwNI) fragment from ISLrh	This study
pMJM79	Em^R^, made from pTRK830 by removing the 1–5 (XhoI-BbsI) fragment from ISLrh	This study
pMJM80	Em^R^, made from pTRK830 by removing the 1–6 (SacI-AlwNI) fragment from ISLrh	This study
pMJM81	Cm^R^ and Em^R^, insert the entire ISLrh in pGK12 at *SacI* site	This study
pMJM82	Cm^R^ and Em^R^, insert the 1–2 fragment in pGK12 at *SacI* site	This study
pMJM90	Cm^R^ and Em^R^, insert the IR2 in pGK12 at *SacI* site	This study
pMJM83	Cm^R^ and Em^R^, insert ISLrh without IRs in pGK12 at *SacI* site	This study
pMJM84	Cm^R^ and Em^R^, insert the 3–4 fragment in pGK12 at *SacI* site	This study
pMJM85	Em^R^, insert the entire ISLrh in pGK12 at *NcoI* site	This study
pMJM86	Em^R^, insert the 1–2 fragment in pGK12 at *NcoI* site	This study
pMJM87	Em^R^, *gusA3* as a reporter gene cloned into pTRK830	This study

To further validate this hypothesis, we amplified the entire ISLrh, the 1–2 fragment, and IR2 from pTRK830 and introduced them to the *SacI* site (5′-GAGCTC-3′) of pGK12 (Fig. 5C), which is immediately adjacent to the original ISLrh insertion site (5′-TGA-3′) in pTRK830 (Fig. 2B). This process generated plasmids pMJM81, pMJM82, and pMJM90, respectively (Table 4; Fig. 5C). Results showed that pMJM81 (10^4^–10^5^ CFU/µg) and pMJM82 (~10^3^ CFU/µg) exhibited higher transformation efficiency in LGG compared to pMJM90, which displayed notably low transformation efficiency (~10^1^ CFU/µg). Furthermore, we also tested DNA fragments without IRs by introducing ISLrh without IRs and the 3–4 fragment (the middle part of ISLrh) into the *SacI* site of pGK12, generating pMJM83 and pMJM84, respectively (Table 4; Fig. 5C). However, no colonies were observed when pMJM83 or pMJM84 was transformed into LGG. These results indicate that the newly obtained replication ability observed in derivatives of pGK12 containing ISLrh, the 1–2 fragment, or solely the IR is likely attributable to the presence of the IR rather than the disruption of an unidentified genetic element within this DNA region of the plasmid.

We conceived another hypothesis that the insertion location of IRs was also critical for the replication of pGK12. To test this, we amplified two ISLrh regions, the entire ISLrh and the 1–2 fragment, and inserted them into pGK12 at the *Nco*I site, 1,275 bp upstream of the *Sac*I site, generating pMJM85 and pMJM86, respectively (Table 4; Fig. 5C). As expected, no LGG transformants were observed when the inserts were introduced at the *Nco*I site, confirming our hypothesis that the insertion location of IRs is critical for activating the replication of resulting plasmids in LGG.

In summary, stable replication of plasmid pGK12 in LGG requires at least one of the IRs, and the specific insertion position of the IRs is also crucial.

### Construction of a new heterologous gene cloning and expression vector

**Fig 7:**
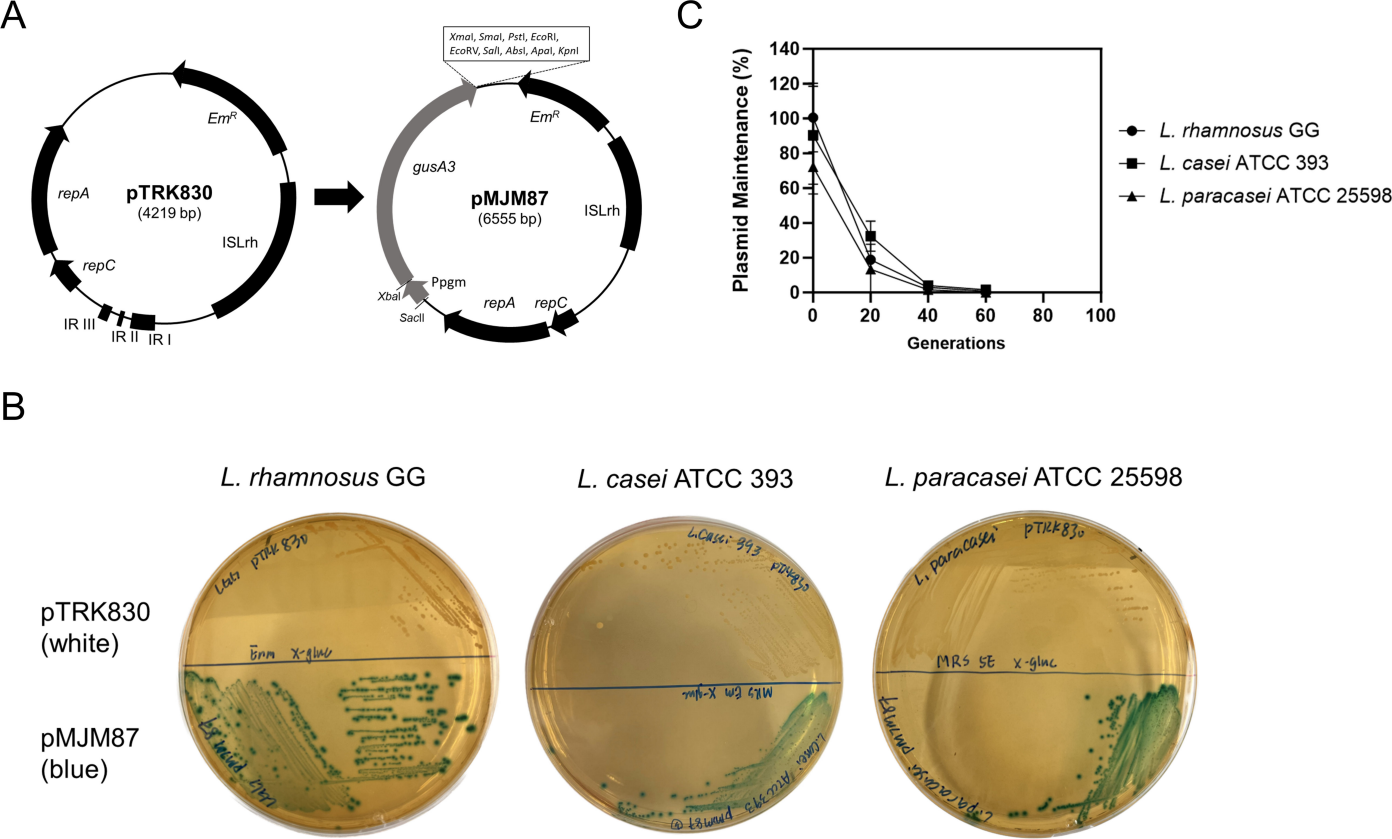
Heterogeneous gene expressing vector construction. (**A**) GusA3 expression cassette was cloned via the *NcoI* site into pTRK830 to construct pMJM87. *Ppgm* is the promoter region of *pgm* gene from *L. acidophilus* NCFM, and *gusA3* gene is the mutant of *L. gasseri* ADH *gusA*. Relative sizes, directions of transcription, and restriction enzyme sites are indicated. (**B**) Lactobacilli strains containing the *gusA3* gene expression vectors turned blue on X-gluc-based MRS agar plates, while those containing control vector pTRK830 remained white. (**C**) Segregational stability of plasmid pMJM87 in lactobacilli strains. Data are presented as means ± SD from at least two biological replicates.

Plasmid pTRK830 was used to express a heterologous gene in new host strains, including LGG, *L. casei* ATCC 393, and *L. paracasei* ATCC 25598, which could not be transformed with pGK12. β-glucuronidase (GUS), encoded by the *L. gasseri* ADH *gusA* gene, is widely used as a reporter gene in lactobacilli strains (25). The *gusA3* gene, a mutant of *gusA*, produces β-glucuronidase with increased activity in neutral pH ranges (26). To construct the *gusA3* gene expression vector, we used the promoter of phosphoglycerate mutase (*Ppgm*) from *L. acidophilus*, resulting in the expression vector designated pMJM87 (Fig. 7A). After electroporation with pMJM87, blue colonies appeared on selective de-Man-Rogosa-Sharpe (MRS) agar plates with X-gluc (Fig. 7B), indicating successful expression of the *gusA3* gene in all three strains.

We then assessed the segregational stability of plasmid pMJM87 in these three host strains (Fig. 7C). The results showed that pMJM87 (Fig. 7C) was less stable than pTRK830 (Fig. 3) under non-selective conditions in all three host strains, with most of the populations losing pMJM87 after 40 generations. This suggests that antibiotics are essential for pMJM87 maintenance in these strains. Additionally, the transformation efficiency of pMJM87 in these strains is about 10 times lower than that of pTRK830. The reduced plasmid segregational stability of pMJM87 can be attributed to the decreased stability of RCR plasmids as their size increases (27, 28). In conclusion, pTRK830 can serve as an effective gene cloning and expression vector for heterologous gene expression in various lactobacilli strains.

## DISCUSSION

**Fig 8:**
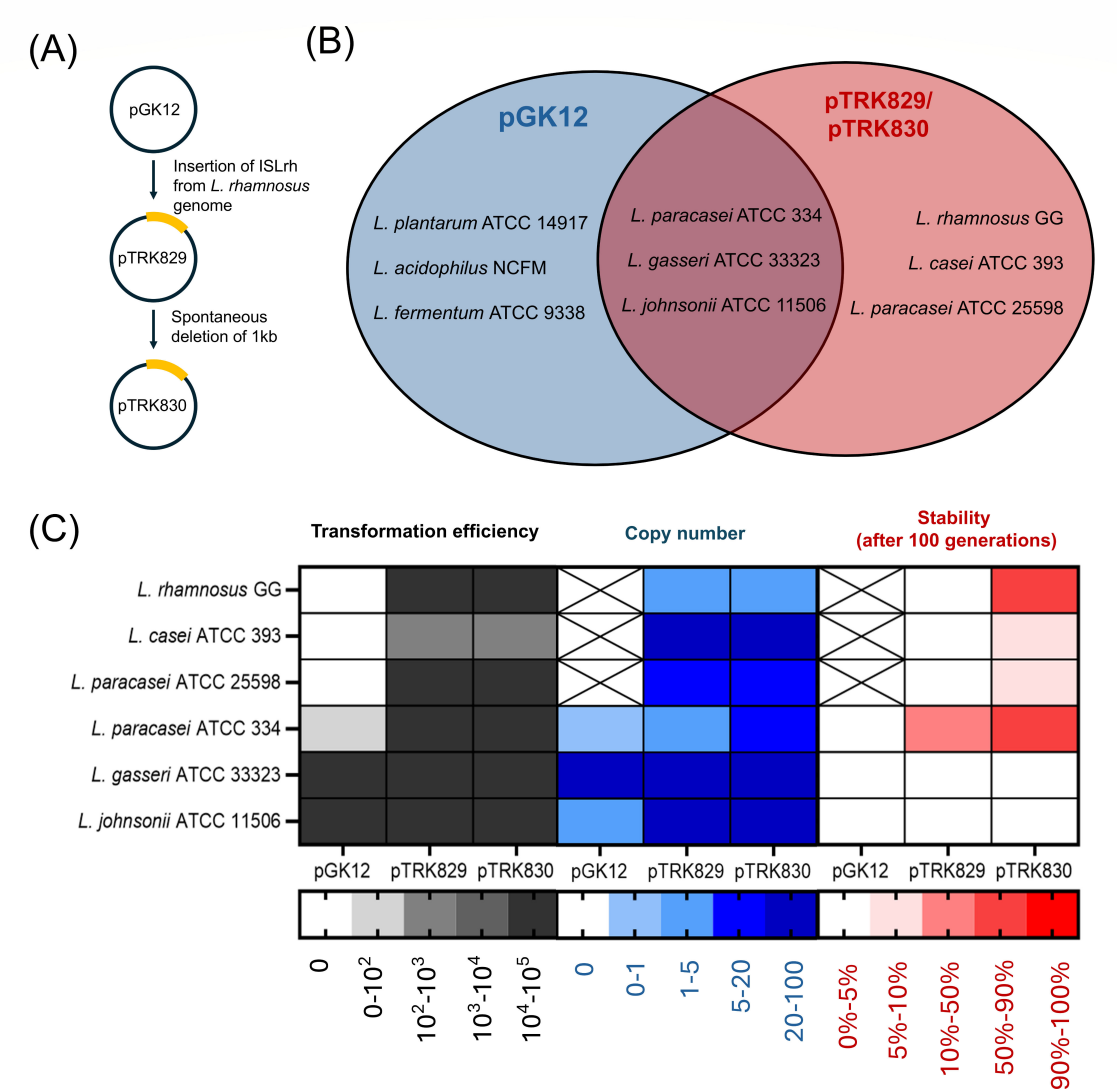
Summary of pGK12 and its ISLrh-inserted variants across lactobacilli strains. (**A**) Schematic representation of pGK12 acquiring ISLrh (yellow arc rectangle) from the *L. rhamnosus* genome, leading to the formation of new variants, pTRK829 and pTRK830. (**B**) Venn diagram summarizing the impact of ISLrh insertion on the plasmid’s host range. The insertion of ISLrh changes the plasmids’ host range, expanding transformability to new strains (red area), retaining transformability in some strains (overlap area), and losing transformability in others (blue area). (**C**) Heat map summarizing the transformation efficiency, copy number, and stability of pGK12, pTRK829, and pTRK830 across different lactobacilli strains. “X” indicates that the plasmid pGK12 could not be successfully transformed into the respective strain, resulting in no data availability. The data highlight strain-specific differences in plasmid performance, with ISLrh insertions contributing to changed host compatibility and plasmid maintenance.

In this study, we have shown that the transposition of an IS element into a plasmid can alter its effective host range in lactobacilli strains (Fig. 8). We observed that pGK12 obtained an IS element from the *L. rhamnosus* M1 genome by chance. The unique insertion of the IS element, ISLrh, into pGK12 enabled the resulting plasmids, pTRK829 and pTRK830, to replicate in *L. rhamnosus* M1 and related species such as LGG, *L. casei* ATCC 393, and *L. paracasei* ATCC 25598, which were previously non-transformable with pGK12. Additionally, we found that some strains can be transformed with both pGK12 and its ISLrh-inserted variants, while some strains can only be transformed with pGK12 but not its variants. This work expands our understanding of the relationship between plasmids and IS elements.

One of the key observations in this study was that the insertion of ISLrh not only altered the plasmid host range but also affected plasmid stability and copy number. We conducted a comprehensive analysis of different plasmids (pGK12, pTRK829, and pTRK830) across multiple host strains (LGG, *L. casei* ATCC 393, *L. paracasei* ATCC 25598, *L. paracasei* ATCC 334, *L. gasseri* ATCC 33323, and *L. johnsonii* ATCC 11506). Generally, the smaller plasmid, pTRK830, exhibited a higher copy number and greater stability compared to pTRK829 across most host strains, except for *L. gasseri* ATCC 33323 and *L. johnsonii* ATCC 11506. This is likely because the smaller RCR plasmid pTRK830 is replicated more efficiently, resulting in increased copy number and stability. These plasmid stability results should be interpreted cautiously, as low plasmid retention levels may introduce variability due to stochastic effects, such as plasmid loss due to random segregation events that might impact our conclusions.

Interestingly, the effects of ISLrh on plasmid behaviors varied depending on the host strain. For example, the insertion of ISLrh enhanced both plasmid stability and copy number and reduced plasmid burden in *L. paracasei* ATCC 334. In contrast, it had no measurable effect in *L. gasseri* ATCC 33323, and in *L. johnsonii* ATCC 11506, ISLrh increased plasmid copy number but reduced stability and increased plasmid burden. These differences highlight the strain-dependent interaction between ISLrh and host replication systems, underscoring the importance of host-specific factors in determining the impact of IS element insertions on plasmid behavior. However, it is important to note that the observed differences in plasmid behavior may also be influenced by the inherent instability of plasmids in certain strains. For instance, plasmid instability, particularly in *L. gasseri* ATCC 33323 and *L. johnsonii* ATCC 11506, could complicate our analysis of plasmid burden, as plasmids may have already been lost from a significant portion of the population, making it difficult to accurately assess the metabolic impact of maintaining the plasmid.

Another intriguing finding is that the IRs of ISLrh and the insertion location are essential for pGK12 to obtain replication ability in LGG. Specifically, at least one of the IRs of ISLrh is required to be present upstream of the *sso* to achieve replication capacity. Previous studies have proposed two possible reasons for how the insertion of an IS element can change plasmid host range: (i) a promoter within the inserted IS element enables the expression of replication-related genes (29, 30); (ii) the insertion of IS elements disrupts the high fitness cost region of the plasmid, reducing the biological burden imposed by the plasmid (29, 31). However, neither of the above mechanisms can explain our findings. First, we observed that either IR1 or IR2 enables plasmids to acquire replication ability in LGG, suggesting that the sequence direction is not crucial for activating replication. Second, the insertion of mock fragments, such as ISLrh without IRs (pMJM83) and the 3–4 fragment (pMJM84), into pGK12 at the *SacI* site failed to activate plasmid replication in LGG. This indicates that the newly obtained replication capacity resulting from the insertion of ISLrh is not due to improved plasmid fitness from the disruption of any unidentified genetic elements within this DNA region that might contribute to plasmid burden. Based on these findings, we propose a new possible mechanism that ISLrh or the IRs of ISLrh may change the structure or topology of the plasmid origin. This new origin structure changes the plasmid transformation and replication efficiency in different strains, thereby changing the plasmid host range. Further investigations are needed to confirm this hypothesis.

Our study also introduces a new cloning and expression vector for lactobacilli research and application. As an RCR plasmid, pTRK829 was unstable and spontaneously lost 1,019 bp to maintain a smaller size. The smaller plasmid pTRK830 exhibits enhanced stability compared to pTRK829 in their host strains, which could not be stably transformed with pGK12. Our characterization of these two novel plasmids highlights the potential of pTRK830 as a molecular tool for heterogeneous gene cloning and expression in lactobacilli. Moreover, we successfully constructed an expression vector based on pTRK830 to express *gusA3* in various lactobacilli. Cloning of heterologous DNA in RCR vectors can decrease the plasmid stability, particularly with large DNA inserts, as supported by previous studies (32, 33). Consistent with this trend, the expression vector pMJM87 exhibited lower stability compared to pTRK830 under non-selective conditions. Hence, pTRK830 appears to be more suited for constructing vectors with smaller inserts and with maintenance at selective conditions. Moreover, pTRK830 is also suitable for use as a genome editing vector, as it can be easily cured after editing the genome. Despite potential instability problems, it is still reasonable to use the RCR plasmid pTRK830 for developing cloning and expression vectors in various species lacking other genetic and biotechnological tools.

The high genomic diversity among lactobacilli strains poses challenges in identifying plasmids that are compatible with a wide range of strains (34, 35). Typically, when attempting genetic manipulation in a new host, the first step is to test the replication capability of existing shuttle vectors. If no suitable vectors are available, it becomes necessary to construct new ones by adapting replicons from the cryptic plasmids of the target strain. Moreover, bacterial defense systems, such as restriction-modification systems and CRISPR systems, hinder the uptake of foreign plasmid DNA (36, 37). Plasmid methylation or mutation of restriction enzyme sites or CRISPR recognition sites could be another possible way to construct suitable plasmids. Our findings provide an additional method for developing new stable vectors by exploiting IS elements, or fractions thereof, to activate the replication ability of a plasmid in previously incompatible host strains.

## MATERIALS AND METHODS

### Bacterial strains and growth conditions

The lactobacilli strains and plasmids used in this study are listed in Tables 1 and 4, respectively. Lactobacilli were grown in MRS broth or on 1.5% agar plates (Hardy Diagnostics, USA) at 37°C under anaerobic conditions. *E. coli* MC1061 was cultured in Luria-Bertani medium at 37°C with vigorous shaking. Antibiotics were added to the growth medium when applicable: erythromycin (Em) 5 µg/mL for lactobacilli strains and 150 µg/mL for *E. coli*.

### DNA manipulations and molecular cloning

The genomic DNA of lactobacilli was extracted using the Promega Wizard genomic DNA purification kit (Promega, USA). A QIAGEN plasmid mini-prep kit (QIAGEN, USA) was used to extract plasmid DNA from *E. coli* cells. DNA restriction enzymes, Klenow fragment, and T4 DNA ligase were purchased from NEB (New England Biolabs, USA). All these enzymes were used following the instructions of the manufacturers. All PCRs were performed according to standard procedures using Taq DNA polymerase (ThermoFisher, USA) or Phusion DNA polymerase (NEB, USA). PCR primers were synthesized by Integrated DNA Technologies and are described in Table 5. PCR products were electrophoresed in a 1.0% agarose gel. DNA fragments were purified using a QIAquick PCR purification kit (QIAGEN, USA) or extracted from agarose gel using a QIAquick gel extraction kit (QIAGEN, USA) prior to downstream application. DNA sequence analysis was performed at the UIUC Core Sequencing Facility (Urbana, IL, USA) using the Sanger sequencing method, and whole plasmid sequencing was performed by the Plasmidsaurus service certified by Oxford Nanopore Technologies.

**TABLE 5:** Primers used in this study^*a*^

Primer	Sequence	Source or reference
IS-F	5′-GTTTGGTTGATAATGAACTGTGCTGATTAC-3′	This study
IS-R	5′-ATTCAATAATCGCATCCGATTGCAG-3′	This study
IS-SacI-1	5′-TATAGCACGAGCTCTGAGAACCTGTCTAG-3′	This study
IS-SacI-2	5′-ACGCGAGCTCGCGCTCGAGAAATATTGCTTGGATAATCTG-3′	This study
IS-SacI-3	5′-ACGCGAGCTCGAACCTGTCTAGTATCTTTTAAGAACTATCTTCAGGAAGG-3′	This study
IR-F	5′-CAGATACTAGACAGGTTCGAGCT-3′	This study
IR-R	5′-CGAACCTGTCTAGTATCTGAGCT-3′	This study
IS-NcoI-1	5′-ATCGCCATGGCTGAGAACCTGTCTAGTATCTGCTGAATCTG-3′	This study
IS-NcoI-2	5′-ATCGCCATGGGCGCTCGAGAAATATTGCTTGGATAATCTG-3′	This study
IS-NcoI-3	5′-ATCGCCATGGGAACCTGTCTAGTATCTTTTAAGAACTATCTTCAGGAAGG-3′	This study
IS-noIR-SacI-F	5′-ATGCGAGCTCGCTGAATCTGGTAGAATTGTGGAAAACCG-3′	This study
IS-noIR-SacI-R	5′-ATGCGAGCTCTTTAAGAACTATCTTCAGGAAGGCCAATACAACC-3′	This study
IS-PN-SacI-F	5′-ATGCGAGCTCCGACAGCTGATTCGCTATTAGAAAGG-3′	This study
IS-PN-SacI-R	5′-ATGCGAGCTCAGCCAATGCACCTGAACGATCTG-3′	This study
Lrh_GroEL_F	5’- ACGGTCGAAGTCGCTGGTC-3′	This study
Lrh_GroEL_R	5’- GGCCGTGCAGGCAAGGTTAC-3′	This study
Lc_GroEL_F	5’- ACGATCGAAGTCGCTGGTTGTG-3′	This study
Lpc_GroEL_F	5’- ACGGTCAAAGTCACTGGTCGTG-3′	This study
Lpc334_GroEL_F	5’- ACGGTCGAAGTCACTGGTCGTG-3′	This study
Lpc_GroEL_R	5’- GAACAATTGGGTCGTGCCGG-3′	This study
Lg_GroEL_F	5’- GGTGCTGGCTCAAAGGATGCAA-3′	This study
Lg_GroEL_R	5’- CAGCAACACCACCAGCAAGCT-3′	This study
Lj_GroEL_F	5’- GTTGAAGGTGCTGGTTCTAAGGAAGC-3′	This study
Lj_GroEL_R	5’- GACAGCAACACCACCAGCAAG-3′	This study
Erm_F	5’- AGGATGAAAATATTCTCTTGGAACC-3′	This study
Erm_R	5’- ATCGTGGAATACGGGTTTGCT-3′	This study

^*a*^ Introduced restriction sequences are underlined.

### Electroporation transformation

The transformation method was optimized based on a previous study (38). A volume of 100 µL from an overnight culture was inoculated in 10 mL prewarmed MRS medium and incubated without agitation at 37°C. After overnight growth, 2 mL of the culture was inoculated into 100 mL of freshly prepared, prewarmed MRS medium, and incubated at 37°C until its optical density (OD_600_) reached 0.5–0.7. Cells were harvested by centrifugation at 4,000 rpm for 15 min. The cells were washed three times with cold 3.5× sucrose magnesium electroporation buffer containing 1 M sucrose, 3.5 mM MgCl_2_, and pH 7.0. After washing, the cells were resuspended in 1 mL of the same buffer, ready for electroporation. For each transformation, 100 µL cells were mixed with 400 ng of plasmid DNA and incubated on ice for 10 min. DNA concentrations of plasmids used for transformation were quantified using a spectrophotometer (NanoDrop2000c, Thermo, USA). The mixture was then transferred to a 2 mm cuvette (Bio-Rad, USA), and electroporation was performed at 2.5 kV, 200 Ω, and 25 µFD, using a Gene Pulser (Bio-Rad, USA). Then, 900 µL pre-warmed MRS broth was added to the cuvette and transferred to 2 mL pre-warmed MRS broth. The transformed cells were allowed to recover by incubation at 37°C overnight. After overnight recovery, the cells were plated on MRS agar plates with appropriate antibiotics and incubated at 37°C in an anaerobic chamber for 2 days. Transformation rates were expressed as the number of transformants per microgram of plasmid DNA.

### Host range and transformation efficiency

A quantity of 400 ng of pGK12, pTRK829, and pTRK830 was used to transform *L. rhamnosus* M1, *L. rhamnosus* GG, *L. casei* ATCC 393, *L. paracasei* ATCC 25598, *L. paracasei* ATCC 334, *L. gasseri* ATCC 33323, *L. johnsonii* ATCC 11506, *L. paraplantarum* ATCC 700211, *L. plantarum* ATCC 14917, *L. fermentum* ATCC 9338, and *L. acidophilus* NCFM individually. The host range and transformation efficiency of pGK12, pTRK829, and pTRK830 were determined by calculating the number of CFU on selective MRS agar plates.

### Plasmid stability

The segregational stability of pGK12, pTRK829, and pTRK830 was tested in six different LAB strains (LGG, *L. casei* ATCC 393, *L. paracasei* ATCC 25598, *L. paracasei* ATCC 334, *L. gasseri* ATCC 33323, and *L. johnsonii* ATCC 11506) under non-selective conditions. Briefly, plasmid-bearing strains were grown overnight in an MRS medium supplemented with Em (0 generation). Then, 0.1% of the cultures were then inoculated into 10 mL of fresh MRS broth without antibiotics twice a day (every 12 hours) for 5 days (~100 generations). For *L. paracasei* ATCC 334 with pGK12, which grew slowly even in non-selective conditions, 0.1% of the overnight cultures were inoculated into 10 mL of fresh MRS broth without antibiotics once a day for 6 days (~60 generations). Periodically, culture broths were serially diluted and plated on MRS agar plates with and without Em for viable cell counts, and we followed the 25–250 counting rule for enumeration (39). The ratio of CFU on the selective vs non-selective MRS agar plates was used to estimate the stability of different plasmids. The stability of each plasmid in each strain was assessed in at least two biological replicates. At low plasmid retention levels, there is an increased chance of variability due to stochastic effects, such as plasmid loss due to random segregation events, and this should be considered when interpreting the results.

### Determination of plasmid copy number by quantitative real-time PCR

All primers used for real-time qPCR are listed in Table 3. DNA was isolated from three independent stationary phase cultures of each transformant and analyzed as independent replicates throughout the real-time PCR procedure. We defined the stationary phase based on consistent optical density readings, indicating that all samples were in a similar growth phase. All real-time qPCR amplifications were performed using PowerUp SYBR Green Master Mix (Fisher, USA) (40). qPCRs were prepared in triplicate for both chromosomal (*groEL*) and plasmid (*erm*) amplicons. The threshold cycle number (*C_t_*) was determined using a PCR thermocycler (QuantaBio Q, USA) and was used to calculate the relative gene copy number (*N*_relative_) for each plasmid in relation to the chromosomal copy number with the formula $N\text{relative}=2^{\left(C\text{t, groEL}-C\text{t, erm}\right)}$ (41), where Ct, groEL was the *C_t_* for the reaction with *groEL*, and Ct, erm was the *C_t_* for the reaction with *erm*. The plasmid copy number was then classified as low, medium, or high when the copies of plasmids per chromosome were less than 5 copies, ~10 copies, or more than 20 copies, respectively.

### Growth rate analysis

The maximum growth rate of *L. paracaei* ATCC 334, *L. gasseri* ATCC 33323, and *L. johnsonii* ATCC 11506, either lacking plasmids or containing pGK12 and its variants, was compared as follows. Cultures without plasmids were passaged twice and grown to the stationary phase in MRS broth without antibiotics, while cultures containing plasmids were passaged twice and grown to the stationary phase in MRS broth supplemented with erythromycin. Three independent cultures were prepared for each strain, with three replicates per culture. After reaching the stationary phase, the cultures were inoculated 1% into 200 µL of fresh MRS without antibiotics in a 96-well plate. The plate was incubated at 37°C without agitation for 18 h in FilterMax F5 Multi-Mode Microplate Reader (Molecular Devices, USA). The optical density at 595 nm (OD_595nm_) in each well was recorded every 20 min, and the maximum specific growth rate (µ_max_) was defined as the slope of the tangent line at the inflection point of the logistic regression curve and was reported per hour (42).

### Localization of functional regions in ISLrh

Mutations in either *orfA* or *orfB* were created by digestion (XhoI or NheI), blunting (Klenow fragment), and relegation (T4 DNA ligase) to yield plasmid pTRK868 or pTRK869. The presence of the mutation was confirmed by restriction enzyme digestion and sequencing.

Further deletions in ISLrh in pTRK830 were made using the same approach (digestion, blunting, and relegation) using various combinations of SacI, XhoI, PvuII, NheI, BbsI, and AlwNI to yield pMJM71-80. The construction of deletion derivatives was verified by PCR using primers IS-F/R and restriction enzyme analysis, and the correct clones were further confirmed by sequence analysis.

The entire ISLrh and the 1–2 fragment were amplified from pTRK830 using primers IS-SacI-1/3 and IS-SacI-1/2, respectively. The IR2 was assembled by annealing complementary DNA oligonucleotides, IR-F/R. PCR products and the IR2 annealed DNA product were cloned into pGK12 at the *SacI* site, generating pMJM81, pMJM82, and pMJM90. ISLrh without IRs and the 3–4 fragments were amplified from pTRK830 using primers IS-noIR-SacI-F/R and IS-PN-SacI-F/R, respectively. PCR products were cloned into pGK12 at the *SacI* site, generating pMJM83 and pMJM84. To study the effect of insertion position, the entire ISLrh and the 1–2 fragment were amplified from pTRK830 using primers IS-NcoI-1/3 and IS-NcoI-1/2, respectively. PCR products were cloned into pGK12 at the *NcoI* site, generating pMJM85 and pMJM86. The ligation mixtures were used to transform *E. coli* MC1061. Recombinant plasmids were verified by plasmid isolation and restriction enzyme analysis, and the correct clones were further confirmed by whole plasmid sequence analysis.

### Construction of heterologous gene-expressing vector

The vector pMJM28 (Table 4) was enzyme digested at the *NcoI* site to get the GusA3 expressing cassette (26), which contains *P_pgm_* (promoter region of *pgm* gene from *L. acidophilus* NCFM), RBS (region encoding ribosome-binding site of mucus binding protein Mub from *L. acidophilus* NCFM), and *gusA3* gene (mutant of *L. gasseri* ADH *gusA*). GusA3 expressing cassette was cloned via the *NcoI* site into pTRK830 to construct pMJM87. The GusA3 expressing vectors were transformed into LGG, *L. casei* ATCC 393, and *L. paracasei* ATCC 25598, and transformants were plated on MRS agar supplemented with Em (5 µg/mL) and 100 µg/mL 5-Bromo-4-chloro-1*H*-indol-3-yl β-D-glucopyranosiduronic acid (X-gluc; Gold Biotechnology, USA).

### Statistical analysis

The results were reported as the means of independent experiments ± SD. Statistical significance was determined by analysis of variance and unpaired *t*-test. *P* values less than 0.05 were considered to indicate statistically significant differences. Distinct superscript letters were employed to denote significant differences in tables. Graphs were plotted using the GraphPad Prism (GraphPad Software, USA).
